# 17β-Hydroxysteroid Dehydrogenase Type 14 Is a Predictive Marker for Tamoxifen Response in Oestrogen Receptor Positive Breast Cancer

**DOI:** 10.1371/journal.pone.0040568

**Published:** 2012-07-06

**Authors:** Tove Sivik, Cecilia Gunnarsson, Tommy Fornander, Bo Nordenskjöld, Lambert Skoog, Olle Stål, Agneta Jansson

**Affiliations:** 1 Division of Oncology, Department of Clinical and Experimental Medicine, Faculty of Health Sciences, Linköping University, Linköping, Sweden; 2 Division of Clinical Genetics, Department of Clinical and Experimental Medicine, Faculty of Health Sciences, Linköping University, Linköping, Sweden; 3 County Council of Östergötland, Linköping, Sweden; 4 Department of Oncology-Pathology, Karolinska Institutet, Stockholm, Sweden; Karolinska Institutet, Sweden

## Abstract

**Introduction:**

17β-hydroxysteroid dehydrogenases (17βHSDs) are important enzymes regulating the pool of bioactive steroids in the breast. The current study was undertaken in order to evaluate implications of 17βHSD14 in breast cancer, measuring 17βHSD14 protein expression in breast tumours.

**Methods:**

An antibody targeting the 17βHSD14 antigen was generated and validated using *HSD17B14*-transfected cells and a peptide-neutralising assay. Tissue microarrays with tumours from 912 post-menopausal women diagnosed with lymph node-negative breast cancer, and randomised to adjuvant tamoxifen or no endocrine treatment, were analysed for 17βHSD14 protein expression with immunohistochemistry.

**Results:**

Results were obtained from 847 tumours. Patients with oestrogen positive tumours with high 17βHSD14 expression had fewer local recurrences when treated with tamoxifen (HR 0.38; 95% C.I. 0.19–0.77, p = 0.007) compared to patients with lower tumoural 17βHSD14 expression, for whom tamoxifen did not reduce the number of local recurrences (HR 1.19; 95% C.I. 0.54–2.59; p = 0.66). No prognostic importance of 17βHSD14 was seen for systemically untreated patients.

**Conclusions:**

Using a highly specific validated antibody for immunohistochemical analysis of a large number of breast tumours, we have shown that tumoural expression levels of 17βHSD14 can predict the outcome of adjuvant tamoxifen treatment in terms of local recurrence-free survival in patients with lymph node-negative ER+ breast cancer. The results need be verified to confirm any clinical relevance.

## Introduction

Peripheral intracrine sex steroid synthesis from adrenally derived steroid precursors in target organs is an ongoing event throughout life which becomes exceedingly important when the gonadal sources of sex steroids cease, as is the case for oestrogen synthesis in women after menopause [1]. 17β hydroxysteroid dehydrogenases (17βHSDs) comprise a family of 15 enzymes catalysing the stereospecific oxidation/reduction at position C17 of sex steroids [2], thereby affecting the biological potency of these. Of the 17βHSD enzymes described to date, type 1 has been ascribed most importance in breast cancer. Expression levels and activity of 17βHSD1, which reduces oestrone to oestradiol, is thought to be of relevance for the local elevation of oestradiol levels measured in tumours compared to plasma, observed in breast cancer, and indeed intratumoural levels of this enzyme have been shown to be of prognostic importance in breast cancer [3], [4], [5], [6]. 17βHSD2, which was the second 17βHSD to be cloned and described, efficiently oxidises oestradiol to oestrone and has been shown to act as a protective factor in breast cancer [6]. The present study focuses on the fourteenth member of the 17βHSD enzyme family, 17βHSD14. Relatively little is known about the function of this enzyme, however, similarly to 17βHSD2, it has been shown to oxidise oestradiol [7]. In a previous study examining mRNA of *HSD17B14* in tumours from a Swedish breast cancer material, we found high expression levels of the *HSD17B14*-transcript to be associated with longer disease-free survival among breast cancer patients [8]. Furthermore, breast cancer cells transiently over-expressing *HSD17B14* significantly lowered oestradiol levels compared to mock-transfected cells, suggesting a protective role of this enzyme in which 17βHSD14 may act by lowering intra-tumoural levels of oestradiol available for oestrogen receptor (ER) binding.

The non-steroidal anti-oestrogen tamoxifen has shown great efficacy both as adjuvant and neo-adjuvant treatment for breast cancer patients as a whole, however, not all patients benefit from the treatment. Several mechanisms, including the relative abundance of steroid-converting enzymes such as 17βHSDs, have been suggested as factors important for predicting tamoxifen treatment response [9].

The aim of the current study was to further investigate and validate the concept of 17βHSD14 as a marker for improved clinical outcome in breast cancer. Tumours from breast cancer patients participating in a randomised tamoxifen trial were analysed for 17βHSD14 protein expression using immunohistochemistry.

## Materials and Methods

### Patient characteristics

**Figure 1:**
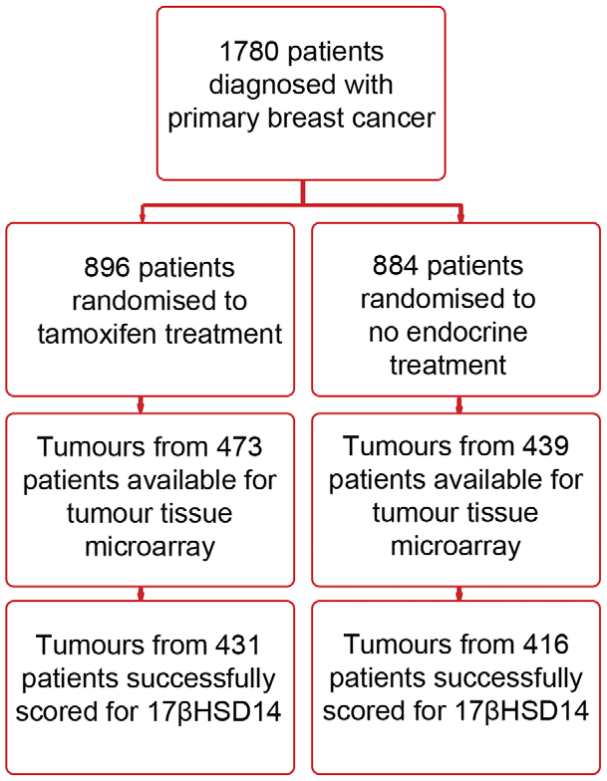
Study design and patient flow chart.

The tumour material in this study was derived from a randomised tamoxifen trial conducted in Stockholm, Sweden 1976–1990 which comprised 1780 low risk breast cancer patients [10]. At the time of diagnosis, all patients were postmenopausal and had lymph node-negative primary breast cancer with tumours of ≤30 mm. Prior to randomization, 432 patients were treated with breast conserving surgery including axillary dissection plus radiation treatment of the breast (50 Gy/5 weeks), whereas the remaining 1 348 patients had a modified radical mastectomy. After surgery, the patients were randomised to tamoxifen treatment (40 mg daily) or no endocrine treatment. After two years of tamoxifen treatment, disease free patients were offered to participate in a trial comparing tamoxifen for an additional three years or no further therapy. The mean follow-up period for patients in the present investigation was 17 years. Loco-regional recurrence was defined as a relapse on the chest wall or in the ipsilateral regional nodes. Information about relapse was supplied by the responsible clinician to the trial centre. Among other deceased patients, follow-up data was collected from regional population registers and the Swedish Cause of Death Registry. A flow-chart of patients included in the initial tamoxifen trial and further included in the current analysis is shown in Fig. 1. The relatively large number of missing tumours is due to logistical and practical problems involved in the recruitment of tumour blocks from the participating trial centers. Patient characteristics compared to the original cohort are shown in Table 1.

**Table 1:** Patient characteristics.

	Patients in present study (n = 912)	Patients with 17βHSD14 expression data (n = 847)	Original Cohort (n = 1780)
**Tumour diameter**			
≤20 mm	697 (79)	647 (79)	1393 (81)
>20 mm	189 (21)	177 (21)	323 (19)
Unavailable	26	23	64
**ER status**			
ER +	686 (77)	633 (77)	1183 (80)
ER −	198 (23)	187 (23)	296 (20)
Unavailable	28	27	301
**PR status**			
PR +	379 (48)	353 (47)	590 (48)
PR −	416 (52)	391 (53)	627 (52)
Unavailable	117	103	563
**Tamoxifen treatment**			
Yes	473 (52)	431 (51)	886 (50)
No	439 (48)	416 (49)	894 (50)

Data presented as number of patients (%).

### Ethics Statement

The trial protocol was approved by the Research Ethics Committee of the Karolinska Institutet. Retrospective tumour analysis, including the current analysis, was approved by the Research Ethics Committee of the Karolinska Institute (dnr 97–451, with amendments). According the approval, informed consent from the patients was not required.

### Tumour material

Samples from 912 patients were available for the present investigation. Sections from formalin-fixed, paraffin-embedded tumour samples collected at surgery were cut and stained with hematoxylin and eosin in order to identify morphologically representative areas. Three cylindrical core tissue specimens (diameter 0.8 mm) were taken (when possible) from each donor block and mounted in a recipient block, with a total amount of at most 243 cores per block. The tissue microarrays (TMAs) were constructed using a manual arrayer (Beecher Instruments, Sun Prairie, WI). TMA blocks were cut in 4 µm sections and mounted on frost-coated slides. ER and progesterone receptor (PR) status of the tumours was originally determined by isoelectric focusing with a cut-off level set to 0.05 fmol/µg DNA. Retrospectively, both ER and PR status of the tumours was examined with immunohistochemistry using the Ventana automated slide stainer (Ventana Medical Systems, Tucson, AZ). The antibodies employed were the monoclonal VentanaMedical Systems' CONFIRM mouse anti-ER primary antibody (clone 6F11) and the monoclonal Ventana Medical Systems' CONFIRM mouse anti-PR primary antibody (clone 16). Comparative analysis of the same tumour material has shown immunohistochemistry and cytosol analysis for the determination of ER-status to be equally effective at predicting long-term effect of adjuvant tamoxifen [11]. In the present study, ER and PR status was derived from immunohistochemical analysis with cut-off levels set to 25% of positively stained tumour cell nuclei. Cytosol measurements were used in cases of missing immunohistochemical data. Immunohistochemistry data on 17βHSD1 and 17βHSD2 expression was previously generated and described [9].

### Antibody production

The peptide sequence (NH2-)CKASRSTPVDAPDIP(-CONH2) corresponding to amino acids 255 to 269 of human 17βHSD14 with an additional terminal cystein residue for affinity purification purposes, was synthesised and sequenced by AgriSera (Vännäs, Sweden). A breed of New Zeeland white rabbits/French lop received initial subcutaneous injections of 200 µg peptide dissolved in a buffer emulsified in Freunds Complete Adjuvant. Three booster injections were administered at 3 months interval; the first including 200 µg peptide and the two following 100 µg. Freunds Incomplete Adjuvant was used with the booster injections. The animals were finally bled one week after the last immunisation and were thereafter sacrificed by injection of an intravenous pentobarbital (Apoteket, Stockholm, Sweden). The anti-17βHSD14 antibody was affinity-purified on a column containing a peptide-coated gel matrix (Ultralink; Thermo Fischer Scientific, Waltham, MA). The preparation of the 17βHSD14 antibody was performed with permission given by the Swedish animal welfare authority (dnr A112-06). Peptide-neutralisation assay as well as immunoblot analysis of *HSD17B14*-transfected breast cancer cells confirmed antibody specificity.

### Immunohistochemistry

TMA slides were deparaffinised in xylene and then hydrated in descending concentrations of ethanol. The sections were thereafter treated in a decloaking buffer (Biocare Medical, Concord, CA) in a decloaking chamber to retrieve antigenicity. The temperature was allowed to reach 120°C at which the heat was turned off. The sections were left in the chamber until 90°C was reached and were thereafter removed from the chamber and allowed to cool to room temperature. To reduce non-specific staining, sections were immersed in phosphate buffered saline (PBS) supplemented with 0.1% bovine serum albumin (BSA) and then incubated with a commercial protein blocking solution (Spring Bioscience, Pleasanton, CA) for 10 min. The tissue sections were incubated with the in-house polyclonal rabbit anti-human 17βHSD14 antibody diluted to 1∶1200 in antibody diluent (DakoCytomation, Glostrup, Denmark) at 4°C over night. After a washing step in PBS/BSA, sections were incubated with EnVision horseradish peroxidase conjugated anti-rabbit polymer (DakoCytomation) for 25 min. The immune reaction was visualised by incubating the samples in a solution containing 3,3-diaminobenzidine chromogen supplemented with hydrogen peroxide for eight min. Sections were briefly counterstained with hematoxylin, dehydrated in ascending concentrations of ethanol and finally mounted. A test for antibody specificity was performed by peptide neutralising assay. Pre-incubation of antibody and 17βHSD14-peptide was done for two hours in room temperature with peptide (10^−6^ M) and 17βHSD14 antibody (1∶600) at a molar ratio of 100∶1 in tris buffered saline with tween (TBST) without blocking reagent. Prior to TMA tissue incubation over night, the pre-incubation solution was added to an equal volume of TBST supplemented with blocking solution. The TMAs were investigated microscopically using a Leica LB30T microscope (Leica Microsystems, Wetzlar, Germany) by two independent investigators blinded to clinical data (TS and AJ) and scored as negative, weak, intermediate or strong. In cases where scoring results differed, a consensus score was reached after re-evaluation. Representative slides were photographed using an Olympus SC20 digital camera (Olympus Europe GmbH, Hamburg, Germany).

### Transient transfection of cells with HSD17B14

ZR75-1 and SKBR3 breast cancer epithelial cells (all from American Type Tissue Culture Collection, Manassas, VA) were cultured in phenol-red free Optimem-I (Invitrogen, Carlsbad, CA) supplemented with 4% foetal bovine serum and incubated at 37°C in 5% CO_2_. For western blot analysis cells were seeded in 6-well cell culture plates at 80% confluence. Twenty-four hours after seeding, the cells were transfected with a commercial *HSD17B14*-plasmid (Origene, Rockville, MD, USA), using Xtremegene transfection reagent (Roche Diagnostics, Indianapolis, IN). Mock-transfected cells were incubated with the same amount of a vector missing the *HSD17B14* insert.

### Western blot

Forty-eight hours post-transfection, lysates of 30 µg of protein from transfected cells were subjected to electrophoretic separation on 5–15% SDS-PAGE gels (BioRad, Hercules, CA). Proteins were transferred to membranes and thereafter blocked in TBST supplemented with 5% non-fat skimmed milk (BioRad). Membranes were incubated with the previously described polyclonal rabbit anti-17βHSD14 antibody, diluted 1∶1000 in blocking solution at 4°C over night. The membranes were then washed and incubated at RT for 60 min. with a secondary HRP-conjugated anti-rabbit IgG, diluted to 1∶2000. Blots were washed three times in TBST and bound antibodies were detected using enhanced chemiluminescence plus detection reagents (Amersham, Arlington Heights, IL). Results were visualised using the LAS1000 CCD-camera detection system (FujiFilm, Tokyo, Japan). A monoclonal anti-β-actin antibody (Cell Signaling, Beverly, MA) was used to control for equal loading.

### Statistical analysis

Relationships between grouped variables were analysed using χ^2^ test. Survival curves were produced according to the lifetable method described by Kaplan and Meier. Differences between curves were estimated using log-rank tests. Analyses of recurrence rates were performed with Cox proportional hazard regression. Tests for interaction between 17βHSD14 and tamoxifen benefit were performed by inclusion of product terms in the model. All the procedures were comprised in the statistical package STATISTICA 9.0 (StatSoft Scandinavia AB, Uppsala, Sweden). All p-values were two sided, and p<0.05 was considered to be statistically significant.

## Results

### Tumour expression of 17βHSD14 protein

**Figure 2:**
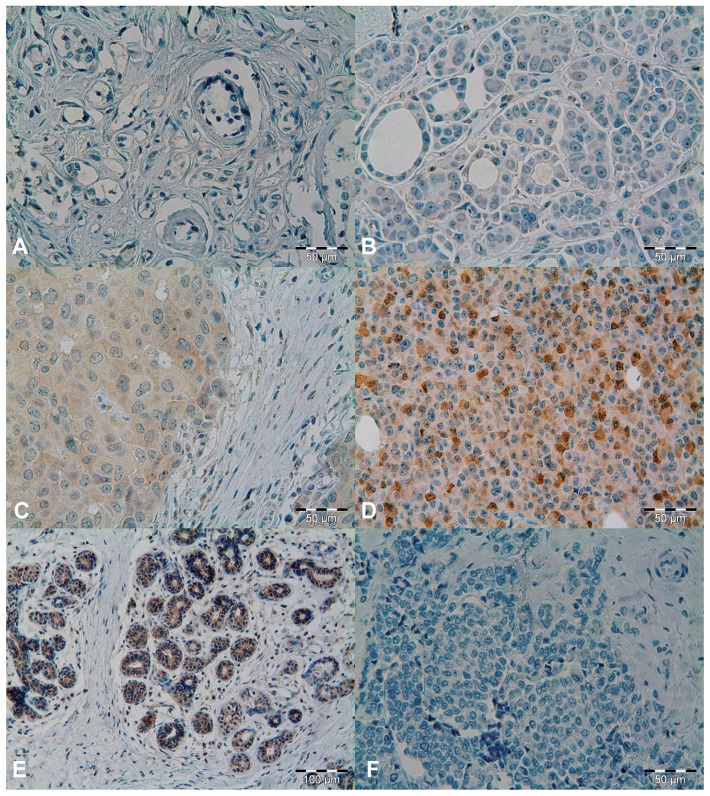
17βHSD14 immunohistochemical staining. Tumour tissue representing (A) negative, (B) weak, (C) intermediate, (D) strong immunopositivity. (E) Breast tissue from healthy individual. (F) Tumour section stained with anti-17βHSD14 antibody pre-incubated with a 17βHSD14 peptide.

**Figure 3:**
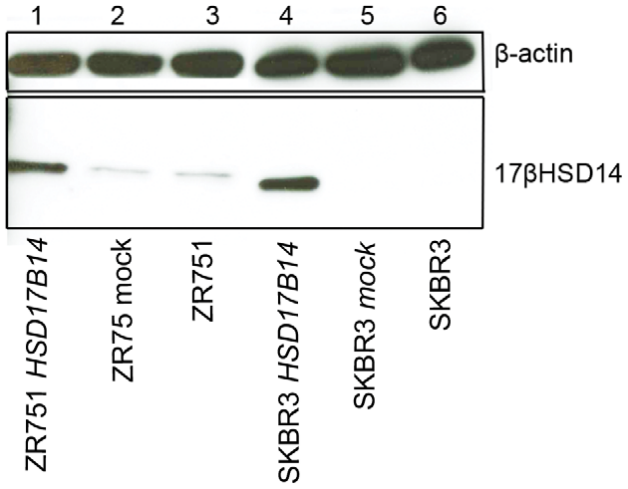
Immunoblot analysis of lysates from ZR75-1 and SKBR3 breast cancer cells. Lower bands represent 17βHSD14 at an estimated size of 28 kDa. In lane 1 and 4 cells transiently over-expressing *HSD17B14*, lane 2 and 5 mock-transfected cells and lane 3 and 6 untreated cells. B-actin serves as a control for equal loading.

Protein expression of 17βHSD14 was analysed in tumours from 912 patients. Of these, 431 patients who received tamoxifen, and 416 who did not receive any endocrine treatment, were successfully scored for 17βHSD14 protein expression in their tumours. When present, staining was exclusively cytoplasmic and graded as negative in 25 (3%) cases, weak in 50 (6%), intermediate in 218 (26%) or strong in 554 (65%) cases (representative images in Fig. 2A–D). A comparative example of 17βHSD14 expression in breast tissue from a healthy donor is seen in Fig. 2E. 17βHSD14 was negatively correlated with progesterone receptor expression (PR) (p = 0.023) and positively correlated with 17βHSD1 (p<0.0001) and 17βHSD2 expression (p<0.0001). There were no associations between 17βHSD14 expression and ER status (p = 0.12) or tumour size (p = 0.64), (Table 2). Specificity of the antibody raised and used for immunohistochemical staining was confirmed by peptide-neutralisation assay (Fig. 2F). The immunoblot analysis (Fig. 3) revealed upregulation of a single band at 28 kDa corresponding to the 17βHSD14 protein in *HSD17B14* transfected cells compared to mock-transfected and non-transfected cells.

**Table 2:** Expression of 17βHSD14 in relation to tumour size, receptor status and 17βHSD1 and 17βHSD2-expression.

	17βHSD14 expression		
	Neg/weak/intermediate	High	Significance (p)
**Tumour size**			
≤20 mm	222 (34)	425 (66)	
≥20 mm	64 (36)	113 (64)	0.64
**ER status**			
ER+	229 (36)	404 (64)	
ER−	56 (30)	131 (70)	0.12
**PR status**			
PR+	140 (40)	213 (60)	
PR−	124 (32)	267 (68)	0.023
**17βHSD1 expression**			
Neg/weak	207 (42)	285 (58)	
Intermediate/ strong	85 (25)	261 (75)	<0.0001
**17βHSD2 expression**			
Neg/weak	219 (41)	312 (59)	
Intermediate/ strong	64 (23)	220 (77)	<0.0001

Data presented as number of patients (%).

### Prognostic and predictive value of 17βHSD14 expression in patients with or without tamoxifen treatment

**Figure 4:**
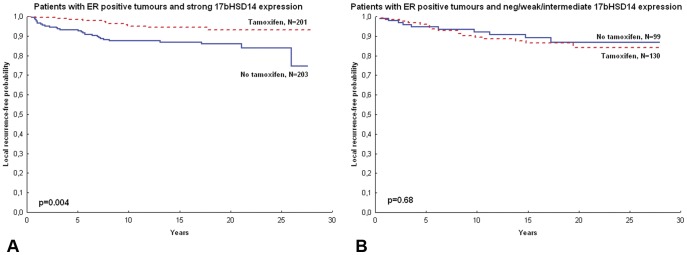
Kaplan-Meier curves depicting local recurrence-free probability in patients stratified by tamoxifen use. (A) Patients with ER+ tumours and strong 17βHSD14 expression. (B) Patients with ER positive tumours and negative/low or intermediate 17βHSD14 expression. ER = oestrogen receptor, 17bHSD = 17β hydroxysteroid dehydrogenase.

**Figure 5:**
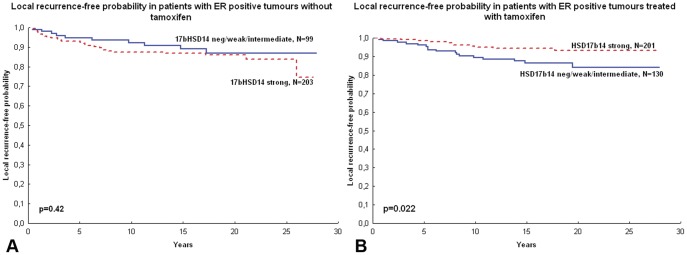
Kaplan-Meier curves depicting local recurrence-free probability in patients stratified by tumoural 17βHSD14 expression. (A) Local recurrence-free probability of patients with ER positive tumours without tamoxifen. (B) Local recurrence-free probability of patients with ER positive tumours treated with tamoxifen. P-values are generated by log-rank tests. ER = oestrogen receptor, 17bHSD = 17β hydroxysteroid dehydrogenase.

For survival analysis, patients were categorised according to intensity of 17βHSD14 staining, where the strongest expression group representing the majority of the material, was compared with patients bearing tumours expressing negative, weak and intermediate levels of 17βHSD14. Among patients with ER positive tumours and a high expression of 17βHSD14, tamoxifen significantly reduced local recurrence rates in univariate Cox models (HR 0.38; 95% C.I. 0.19–0.77, p = 0.007) when compared to patients bearing tumours with intermediate, low or negative expression levels (HR 1.19; 95% C.I. 0.54–2.59; p = 0.66), for whom tamoxifen did not have an impact on local recurrence rates (Fig. 4, Table 3). The interaction between 17βHSD14 and tamoxifen was significant for ER+ patients (p = 0.027). The interaction term stayed significant in a multivariate model also including the established prognostic factors tumour size, PR, and the HSD17B1>HSD17B2 index, for patients with ER positive tumours. There was no significant difference in local recurrence rates in relation to 17βHSD14 expression among systemically untreated patients (p = 0.42), but among tamoxifen treated patients with ER positive tumours, strong 17βHSD14 tumoural expression was associated with significantly better tamoxifen benefit (p = 0.022) (Fig. 5). There were no prognostic or treatment predictive values of 17βHSD14 when considering distant recurrence-free survival or breast cancer-specific survival as end-point (data not shown).

**Table 3:** Cox regression analysis of local recurrence-free probability for breast cancer patients with ER-positive tumours with regards to tumoural 17βHSD14 expression.

Local recurrence-free survival Tamoxifen vs no tamoxifen			
	HR	p	p_interaction_
**17βHSD14 neg/weak/ intermediate**	1.19	0.66	
**17βHSD14 strong**	0.38	0.0067	0.027

## Discussion

In the current study we analysed 17βHSD14 protein levels in a large cohort of tumours derived from a clinical trial where patients were randomised to adjuvant tamoxifen treatment or no endocrine treatment. In the patient group with tumours expressing high levels of 17βHSD14, tamoxifen significantly reduced the number of local recurrences. Among patients with tumours expressing less 17βHSD14, tamoxifen treatment did not influence the local recurrence rates. It thus appears as though expression levels of 17βHSD14 can predict the benefit of tamoxifen in lowering the number of local recurrences. When analysing all patients receiving tamoxifen, high tumour expression of 17βHSD14 significantly lowered local recurrence rates, and the current study thus supports findings from our previous study in which *HSD17B14* transcripts were analysed in a different patient group, all receiving tamoxifen [8]. In that study, which comprised patients with larger tumours with lymph node involvement, higher *HSD17B14* levels were associated with improved clinical outcome.

The function of 17βHSD14 is not fully understood. We and others have shown that the enzyme oxidises potent oestradiol into less bioactive oestrone [7], [8]. Although ovarian oestrogen production ceases at menopause, the supply of oestrogens is maintained through local production from aromatisation of androgens [12], and a high postmenopausal plasma oestradiol level is associated with a subsequent risk of developing breast cancer [13]. In malignant breast tissue, the tissue/plasma ratio is elevated compared with benign breast tissue [14], suggesting higher tumoural aromatase activity and/or higher reductive than oxidative 17βHSD activity. The role of oestrogens for breast cancer growth is evidenced by the success of anti-hormonal treatment regimens, e.g. selective estrogen receptor modulators such as tamoxifen, in improving clinical outcome in the disease. Although the overall benefit from tamoxifen in reducing the risk of breast cancer relapse is clear, with clinical trials demonstrating an approximate 50% reduction in relapse for patients with ER+ tumours [10], [15], a subset of patients fail to respond to the drug, either due to acquired resistance or to an intrinsic insensitivity to tamoxifen [16]. One of several proposed mechanisms underlying failure to respond to tamoxifen involve a situation in which excessive oestradiol levels cannot be counteracted by normal doses of tamoxifen. This scenario has been described in a murine model by Iino et al who demonstrated that, although tamoxifen inhibited the growth of MCF-7 tumours in athymic mice, the tamoxifen inhibitory effect on tumour growth could be partially reversed by increased oestradiol levels [17]. Indeed, a recent meta analysis by the Early Breast Cancer Trialists' Collaborative Group, reported a greater reduction in breast cancer recurrence in trials of higher daily tamoxifen doses when comparing 20, 30, and 40 mg per day [15]. We found high tumoural expression levels of 17βHSD14 to be associated with better tamoxifen response in terms of risk of developing local recurrence, and it is plausible that the oestradiol lowering capacity of 17βHSD14 contributes to this finding.

17βHSD14 expression was correlated with expression levels of 17βHSD1 and 17βHSD2, two highly potent enzymes catalysing the reduction of oestrone and oxidation of oestradiol respectively, for which expression levels have been previously assessed in the current tumour material [9]. Interestingly, in that study, high tumoural expression of 17βHSD2 as compared with 17βHSD1 was significantly beneficial for tamoxifen response when considering distant recurrence and breast cancer survival as endpoint, whereas it was not significant when considering local recurrence as endpoint [9]. In the current study, the situation was the opposite, with 17βHSD14 having an influence on local recurrence but no impact in predicting distant recurrence or breast cancer-specific survival. This discrepancy is rather surprising, as one would expect these enzymes to all influence the same pathways, namely steroid conversion. It thus seems likely that, although expression levels of 17βHSD1, 17βHSD2 and 17βHSD14 are correlated and possibly co-regulated, the role of 17βHSD14 is separated from that of 17HSD1 and 17βHSD2, with 17βHSD14 in addition to oestradiol, also acting upon other substrates. Indications for 17βHSD14 in local recurrence could implicate a role for the enzyme in interactions with the microenvironment. In a recent publication 17βHSD14 is shown to act immunomodulatory, affecting the levels of 5-androstenediol which in turn is shown to be a significant inducer of anti-inflammatory responses mediated by ERβ in the central nervous system [18]. Whether this role of 17βHSD14 is relevant in neoplasia remains to be elucidated.

In conclusion, we have shown that tumoural expression levels of 17βHSD14 can predict the outcome of adjuvant tamoxifen treatment in terms of local recurrence-free survival in patients with lymph node-negative ER+ breast cancer. Although these results should be verified to confirm any clinical relevance, it is clear that a better understanding of the risk of local recurrence facilitate therapeutic decision making. The mechanism underlying effects related to 17βHSD14 remain elusive, however one might speculate that the enzyme lowers oestradiol levels and thereby enhances the anti-proliferative action of tamoxifen. In cases of low oestradiol oxidising enzymes such as 17βHSD14, more clinical benefit would likely be generated by alternative treatments such as enzyme inhibitors of e.g aromatase or inhibitors of reductive 17βHSD-enzymes. The association of 17βHSD14 with local relapse implies that the enzyme, in addition to oestradiol, works upon other steroid or non-steroid substrates, and that these factors have importance for interactions with the microenvironment.
